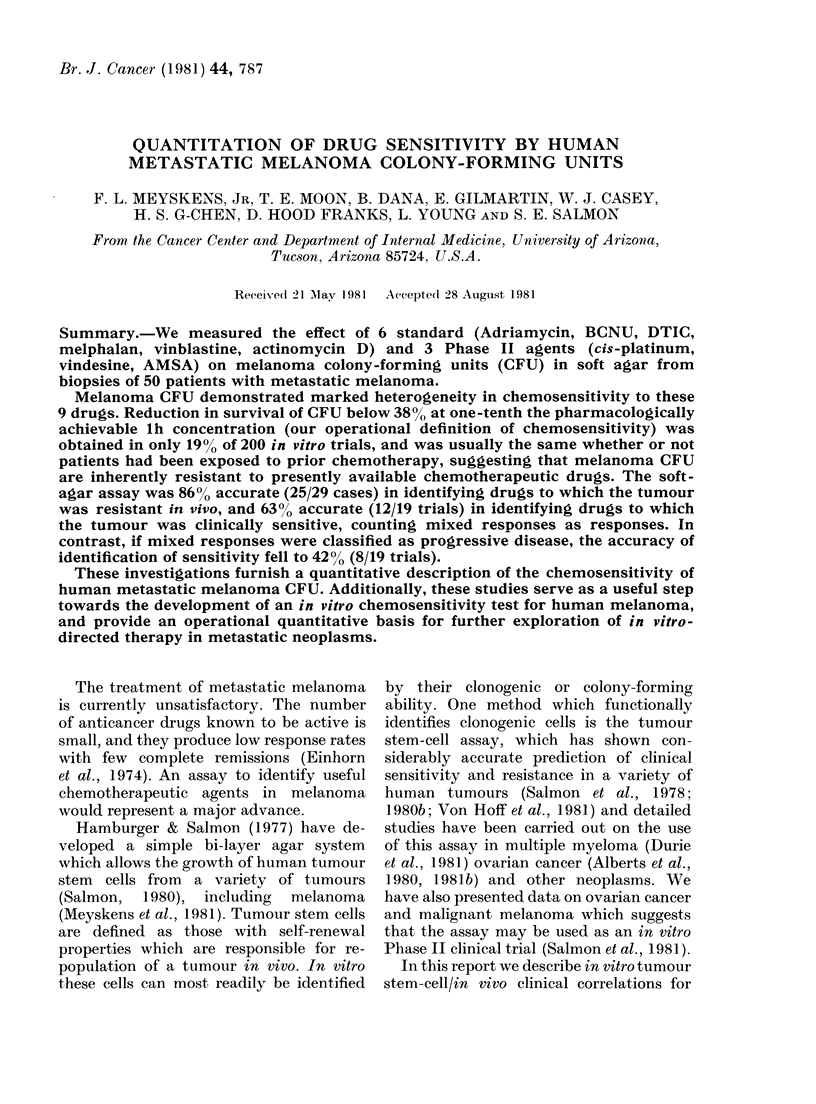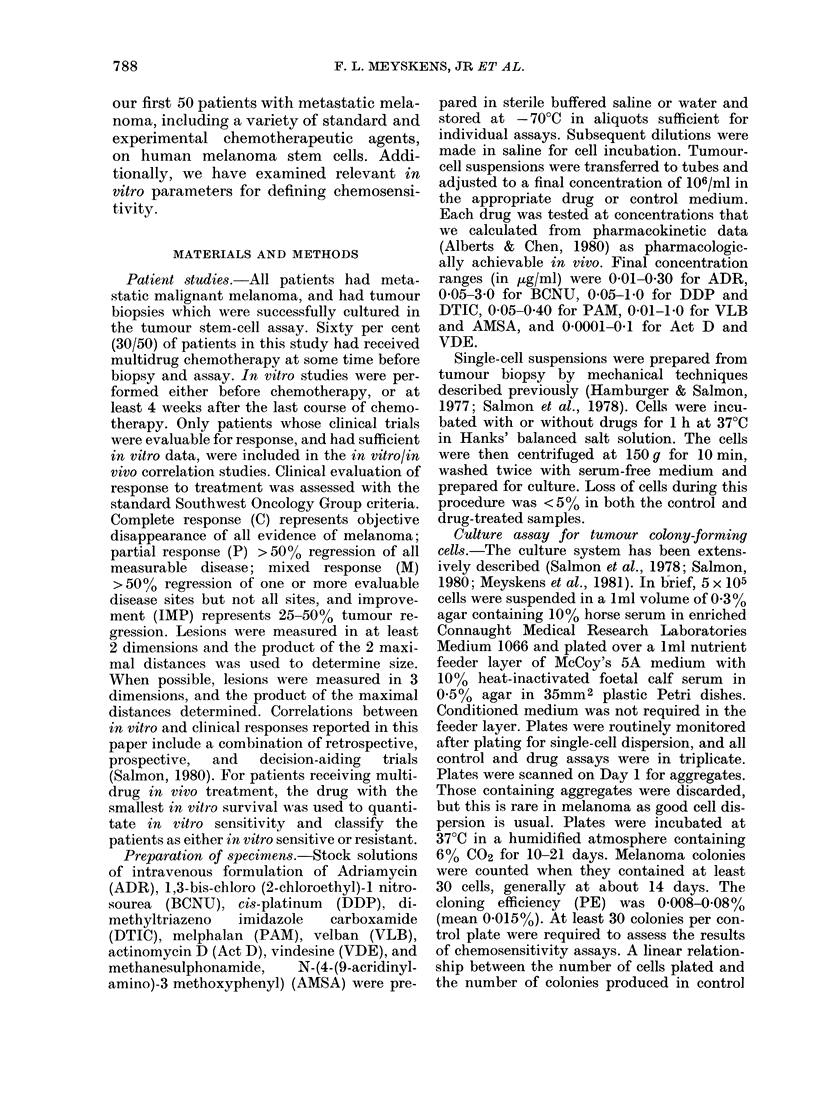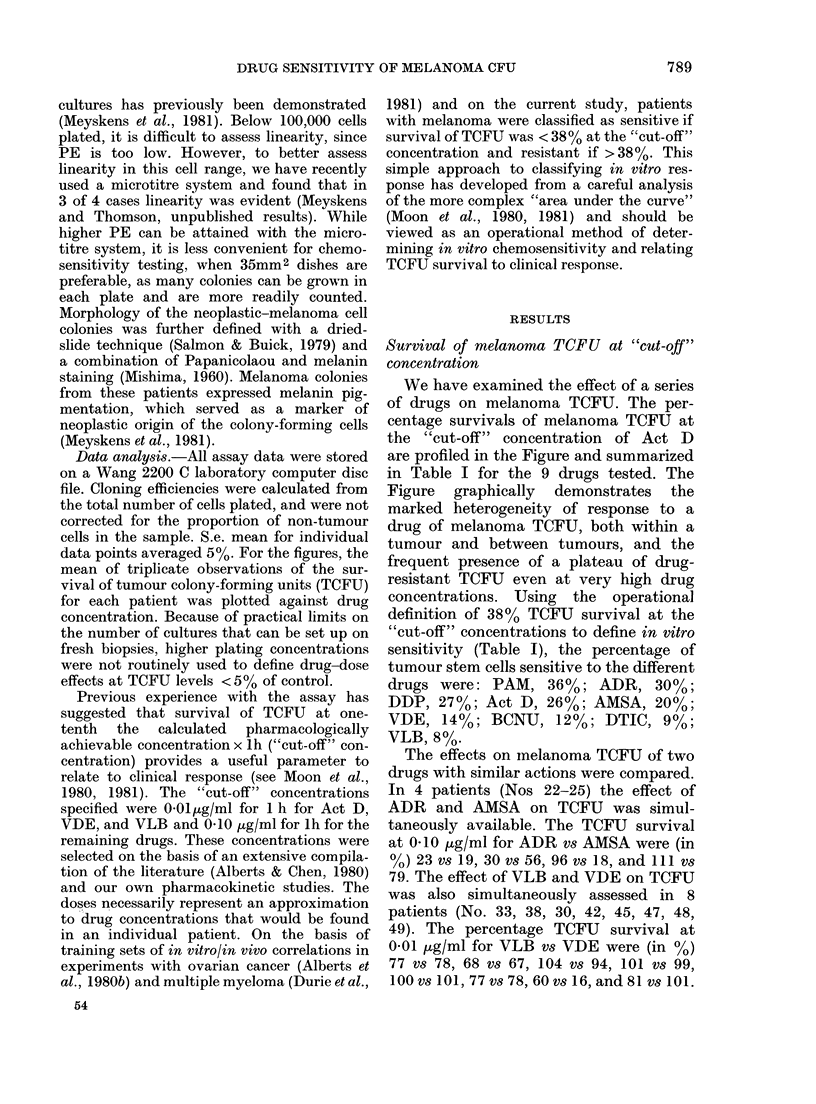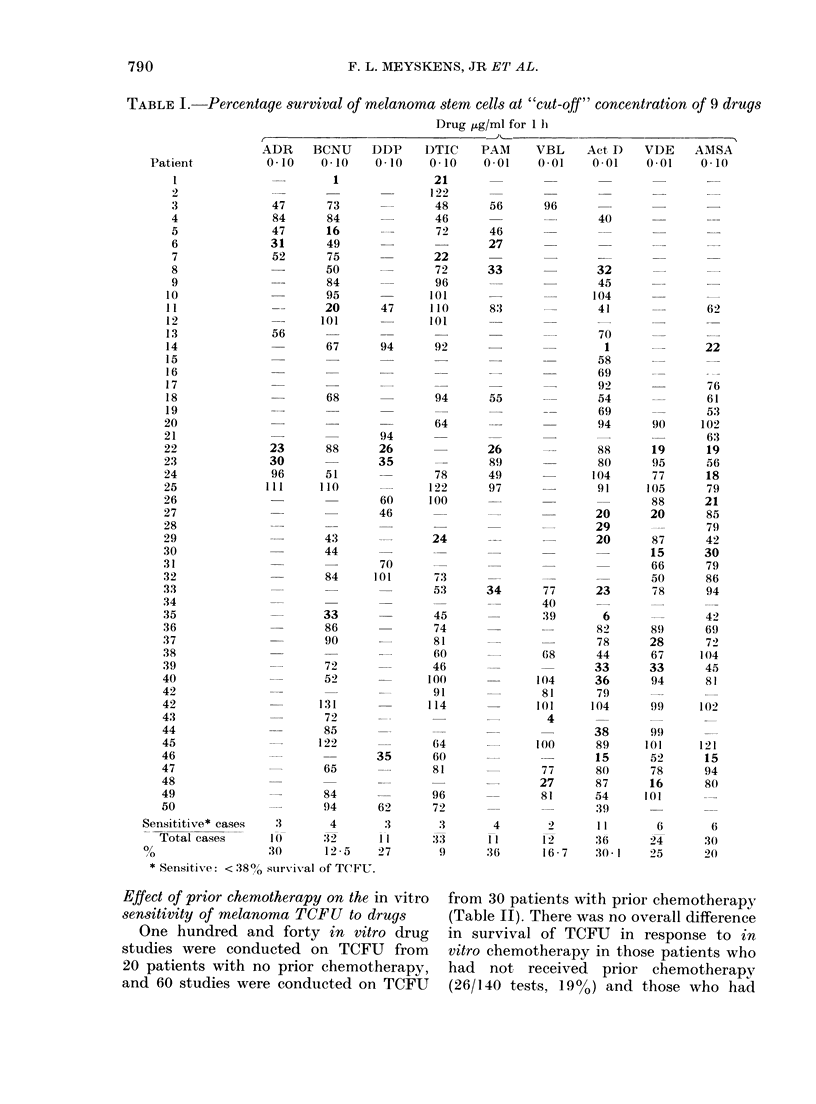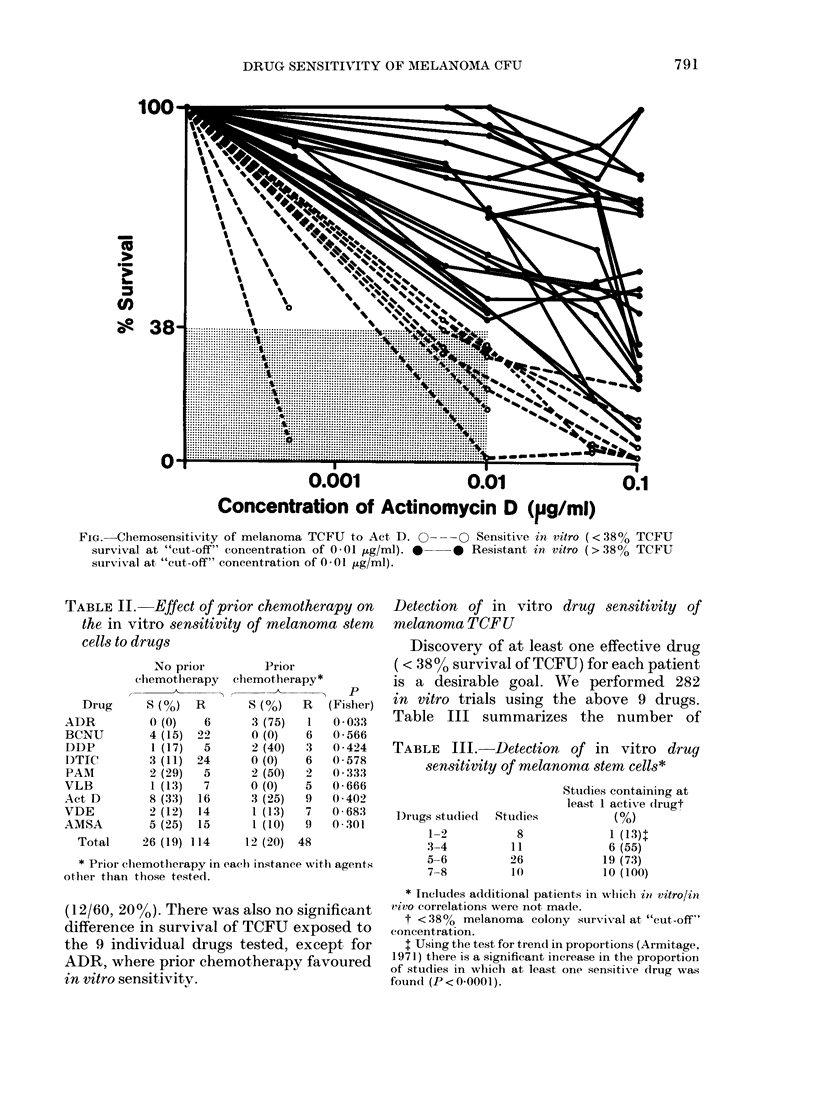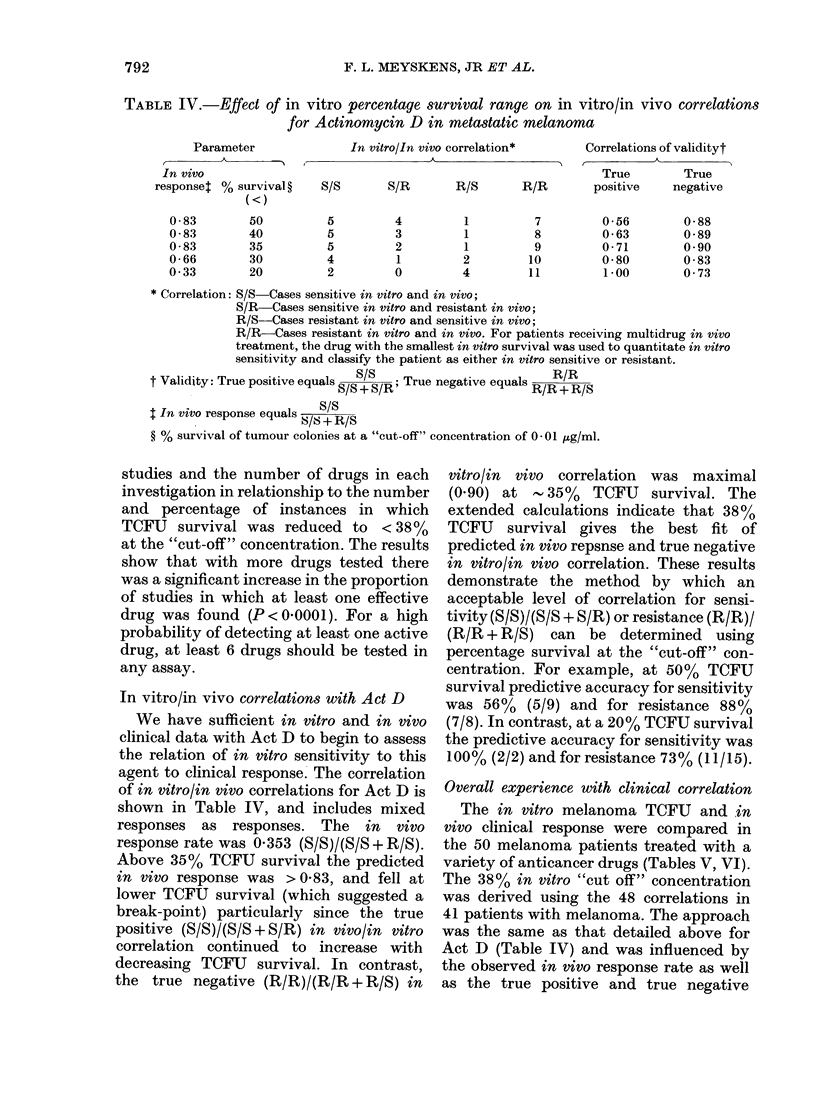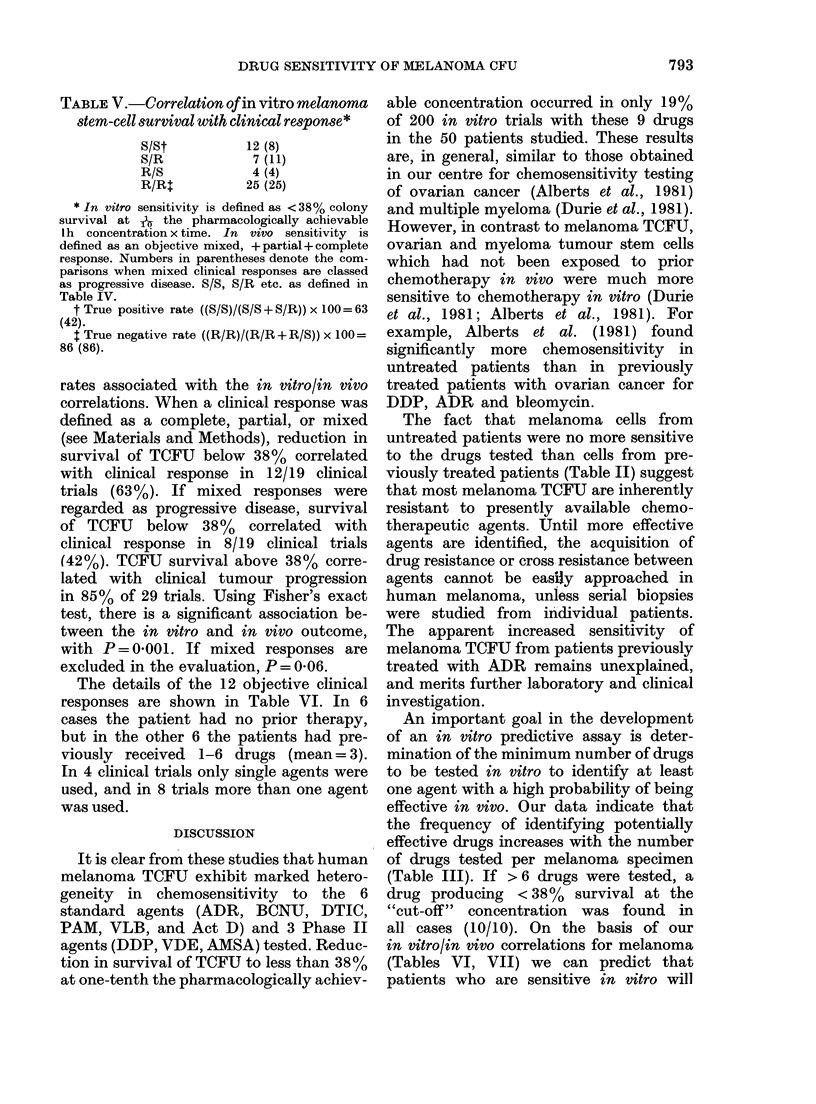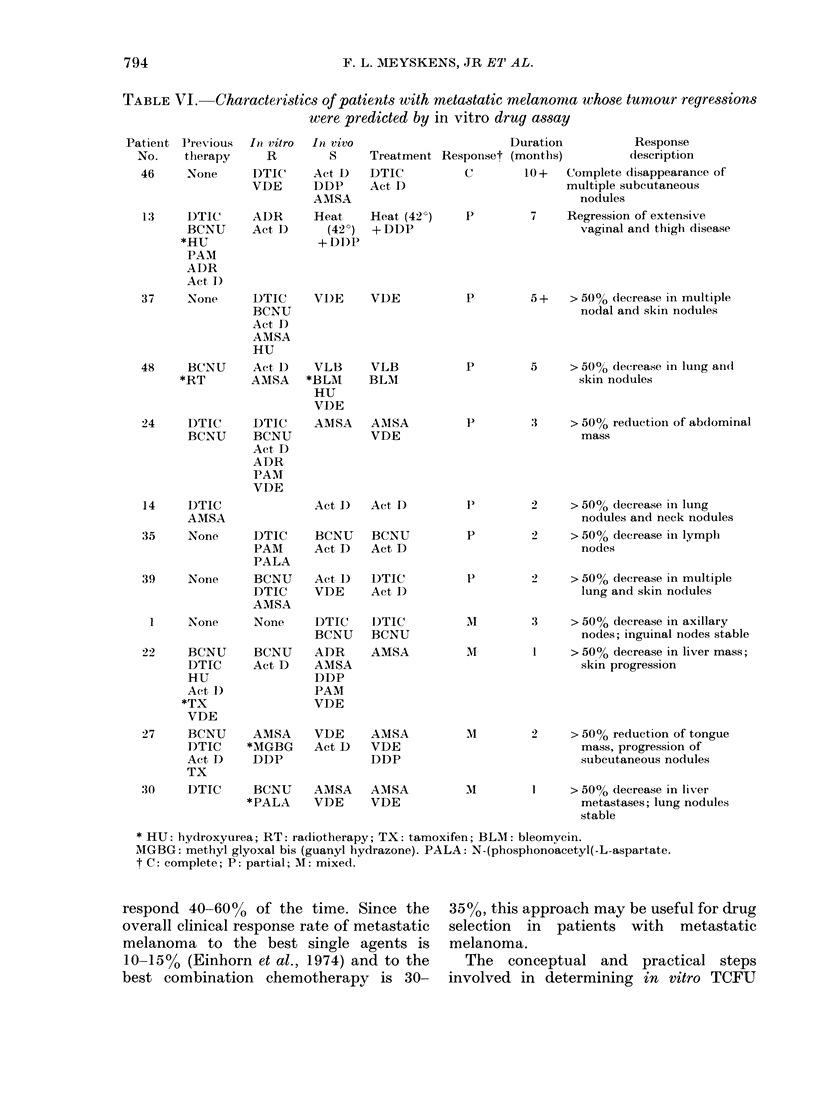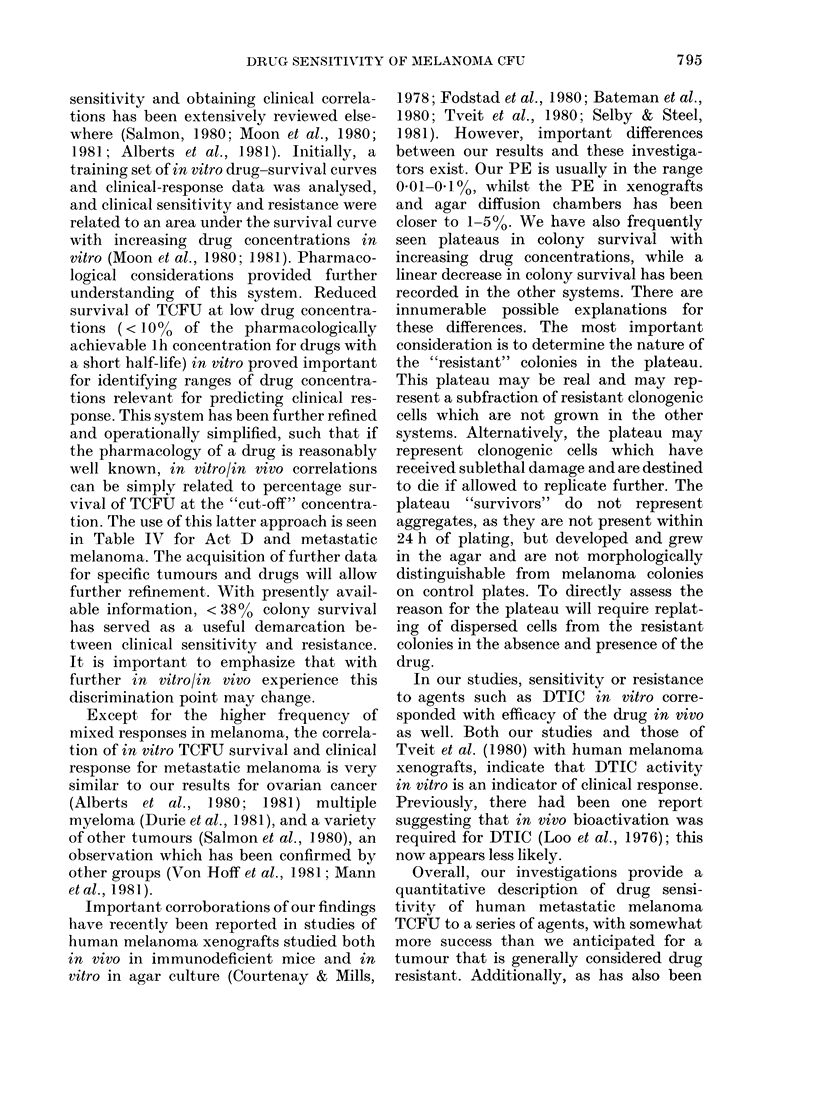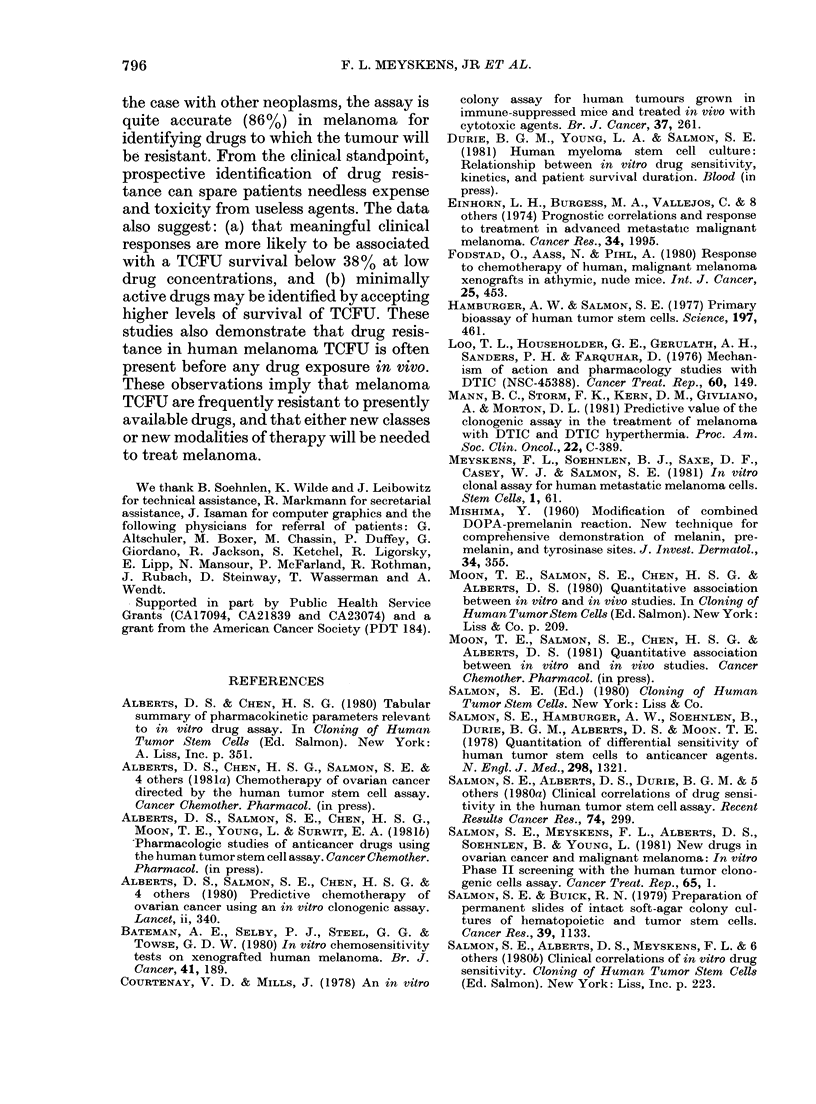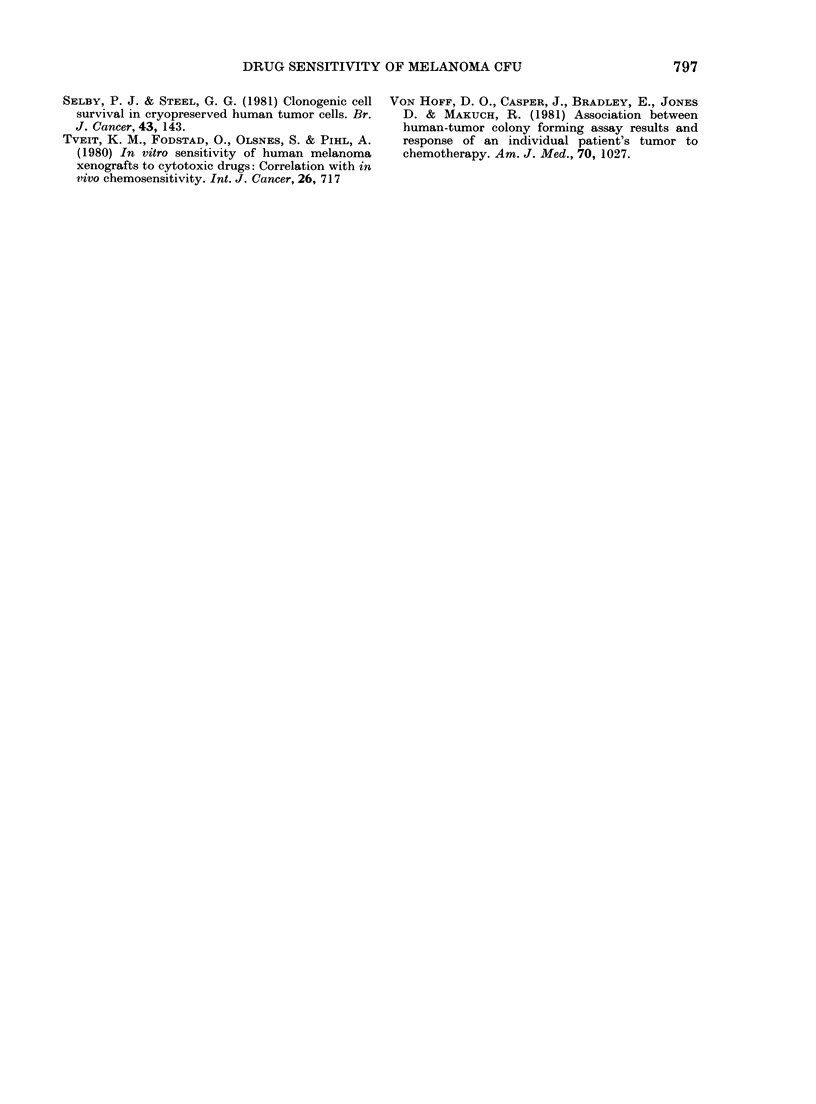# Quantitation of drug sensitivity by human metastatic melanoma colony-forming units.

**DOI:** 10.1038/bjc.1981.277

**Published:** 1981-12

**Authors:** F. L. Meyskens, T. E. Moon, B. Dana, E. Gilmartin, W. J. Casey, H. S. Chen, D. H. Franks, L. Young, S. E. Salmon

## Abstract

We measured the effect of 6 standard (Adriamycin, BCNU, DTIC, melphalan, vinblastine, actinomycin D) and 3 Phase II agents (cis-platinum, vindesine, AMSA) on melanoma colony-forming units (CFU) in soft agar from biopsies of 50 patients with metastatic melanoma. Melanoma CFU demonstrated marked heterogeneity in chemosensitivity to these 9 drugs. Reduction in survival of CFU below 38% at one-tenth the pharmacologically achievable 1h concentration (our operational definition of chemosensitivity) was obtained in only 19% of 200 in vitro trials, and was usually the same whether or not patients had been exposed to prior chemotherapy, suggesting that melanoma CFU are inherently resistant to presently available chemotherapeutic drugs. The soft-agar assay was 86% accurate (25/29 cases) in identifying drugs to which the tumour was resistant in vivo, and 63% accurate (12/19 trials) in identifying drugs to which the tumour was clinically sensitive, counting mixed responses as responses. In contrast, if mixed responses were classified as progressive disease, the accuracy of identification of sensitivity fell to 42% (8/19 trials). These investigations furnish a quantitative description of the chemosensitivity of human metastatic melanoma CFU. Additionally, these studies serve as a useful step towards the development of an in vitro chemosensitivity test for human melanoma, and provide an operational quantitative basis for further exploration of in vitro-directed therapy in metastatic neoplasms.


*Br. J. Cancer* (1981) **44**, 787

### QUANTITATION OF DRUG SENSITIVITY BY HUMAN METASTATIC MELANOMA COLONY-FORMING UNITS

F. L. MEYSKENS, JR, T. E. MOON, B. DANA, E. GILMARTIN, W. J. CASEY, H. S. G-CHEN, D. HOOD FRANKS, L. YOUNG AND S. E. SALMON

*From the Cancer Center and Department of Internal Medicine, University of Arizona, Tucson, Arizona* 85724, *U.S.A.*

Received 21 May 1981 Accepted 28 August 1981

**Summary.—We measured the effect of 6 standard (Adriamycin, BCNU, DTIC, melphalan, vinblastine, actinomycin D) and 3 Phase II agents (*cis*-platinum, vindesine, AMSA) on melanoma colony-forming units (CFU) in soft agar from biopsies of 50 patients with metastatic melanoma.**

**Melanoma CFU demonstrated marked heterogeneity in chemosensitivity to these 9 drugs. Reduction in survival of CFU below 38% at one-tenth the pharmacologically achievable 1h concentration (our operational definition of chemosensitivity) was obtained in only 19% of 200 *in vitro* trials, and was usually the same whether or not patients had been exposed to prior chemotherapy, suggesting that melanoma CFU are inherently resistant to presently available chemotherapeutic drugs. The soft-agar assay was 86% accurate (25/29 cases) in identifying drugs to which the tumour was resistant *in vivo*, and 63% accurate (12/19 trials) in identifying drugs to which the tumour was clinically sensitive, counting mixed responses as responses. In contrast, if mixed responses were classified as progressive disease, the accuracy of identification of sensitivity fell to 42% (8/19 trials).**

**These investigations furnish a quantitative description of the chemosensitivity of human metastatic melanoma CFU. Additionally, these studies serve as a useful step towards the development of an *in vitro* chemosensitivity test for human melanoma, and provide an operational quantitative basis for further exploration of *in vitro*-directed therapy in metastatic neoplasms.**

The treatment of metastatic melanoma is currently unsatisfactory. The number of anticancer drugs known to be active is small, and they produce low response rates with few complete remissions (Einhorn *et al.*, 1974). An assay to identify useful chemotherapeutic agents in melanoma would represent a major advance.

Hamburger & Salmon (1977) have developed a simple bi-layer agar system which allows the growth of human tumour stem cells from a variety of tumours (Salmon, 1980), including melanoma (Meyskens *et al.*, 1981). Tumour stem cells are defined as those with self-renewal properties which are responsible for repopulation of a tumour *in vivo*. *In vitro* these cells can most readily be identified by their clonogenic or colony-forming ability. One method which functionally identifies clonogenic cells is the tumour stem-cell assay, which has shown considerably accurate prediction of clinical sensitivity and resistance in a variety of human tumours (Salmon *et al.*, 1978; 1980*b*; Von Hoff *et al.*, 1981) and detailed studies have been carried out on the use of this assay in multiple myeloma (Durie *et al.*, 1981) ovarian cancer (Alberts *et al.*, 1980, 1981*b*) and other neoplasms. We have also presented data on ovarian cancer and malignant melanoma which suggests that the assay may be used as an *in vitro* Phase II clinical trial (Salmon *et al.*, 1981).

In this report we describe *in vitro* tumour stem-cell/*in vivo* clinical correlations for our first 50 patients with metastatic melanoma, including a variety of standard and experimental chemotherapeutic agents, on human melanoma stem cells. Additionally, we have examined relevant *in vitro* parameters for defining chemosensitivity.

### MATERIALS AND METHODS

*Patient studies.*—All patients had metastatic malignant melanoma, and had tumour biopsies which were successfully cultured in the tumour stem-cell assay. Sixty per cent (30/50) of patients in this study had received multidrug chemotherapy at some time before biopsy and assay. *In vitro* studies were performed either before chemotherapy, or at least 4 weeks after the last course of chemotherapy. Only patients whose clinical trials were evaluable for response, and had sufficient *in vitro* data, were included in the *in vitro/in vivo* correlation studies. Clinical evaluation of response to treatment was assessed with the standard Southwest Oncology Group criteria. Complete response (C) represents objective disappearance of all evidence of melanoma; partial response (P) >50% regression of all measurable disease; mixed response (M) >50% regression of one or more evaluable disease sites but not all sites, and improvement (IMP) represents 25–50% tumour regression. Lesions were measured in at least 2 dimensions and the product of the 2 maximal distances was used to determine size. When possible, lesions were measured in 3 dimensions, and the product of the maximal distances determined. Correlations between *in vitro* and clinical responses reported in this paper include a combination of retrospective, prospective, and decision-aiding trials (Salmon, 1980). For patients receiving multidrug *in vivo* treatment, the drug with the smallest *in vitro* survival was used to quantitate *in vitro* sensitivity and classify the patients as either *in vitro* sensitive or resistant.

*Preparation of specimens.*—Stock solutions of intravenous formulation of Adriamycin (ADR), 1,3-bis-chloro (2-chloroethyl)-1 nitrosourea (BCNU), *cis*-platinum (DDP), dimethyltriazeno imidazole carboxamide (DTIC), melphalan (PAM), velban (VLB), actinomycin D (Act D), vindesine (VDE), and methanesulphonamide, N-(4-(9-acridinylamino)-3 methoxyphenyl) (AMSA) were prepared in sterile buffered saline or water and stored at −70°C in aliquots sufficient for individual assays. Subsequent dilutions were made in saline for cell incubation. Tumour-cell suspensions were transferred to tubes and adjusted to a final concentration of $10^6$/ml in the appropriate drug or control medium. Each drug was tested at concentrations that we calculated from pharmacokinetic data (Alberts & Chen, 1980) as pharmacologically achievable *in vivo*. Final concentration ranges (in μg/ml) were 0·01–0·30 for ADR, 0·05–3·0 for BCNU, 0·05–1·0 for DDP and DTIC, 0·05–0·40 for PAM, 0·01–1·0 for VLB and AMSA, and 0·0001–0·1 for Act D and VDE.

Single-cell suspensions were prepared from tumour biopsy by mechanical techniques described previously (Hamburger & Salmon, 1977; Salmon *et al.*, 1978). Cells were incubated with or without drugs for 1 h at 37°C in Hanks' balanced salt solution. The cells were then centrifuged at 150 *g* for 10 min, washed twice with serum-free medium and prepared for culture. Loss of cells during this procedure was <5% in both the control and drug-treated samples.

*Culture assay for tumour colony-forming cells.*—The culture system has been extensively described (Salmon *et al.*, 1978; Salmon, 1980; Meyskens *et al.*, 1981). In brief, $5 \times 10^5$ cells were suspended in a 1ml volume of 0·3% agar containing 10% horse serum in enriched Connaught Medical Research Laboratories Medium 1066 and plated over a 1ml nutrient feeder layer of McCoy's 5A medium with 10% heat-inactivated foetal calf serum in 0·5% agar in 35mm$^2$ plastic Petri dishes. Conditioned medium was not required in the feeder layer. Plates were routinely monitored after plating for single-cell dispersion, and all control and drug assays were in triplicate. Plates were scanned on Day 1 for aggregates. Those containing aggregates were discarded, but this is rare in melanoma as good cell dispersion is usual. Plates were incubated at 37°C in a humidified atmosphere containing 6% $CO_2$ for 10–21 days. Melanoma colonies were counted when they contained at least 30 cells, generally at about 14 days. The cloning efficiency (PE) was 0·008–0·08% (mean 0·015%). At least 30 colonies per control plate were required to assess the results of chemosensitivity assays. A linear relationship between the number of cells plated and the number of colonies produced in control cultures has previously been demonstrated (Meyskens *et al.*, 1981). Below 100,000 cells plated, it is difficult to assess linearity, since PE is too low. However, to better assess linearity in this cell range, we have recently used a microtitre system and found that in 3 of 4 cases linearity was evident (Meyskens and Thomson, unpublished results). While higher PE can be attained with the microtitre system, it is less convenient for chemosensitivity testing, when 35mm$^2$ dishes are preferable, as many colonies can be grown in each plate and are more readily counted. Morphology of the neoplastic–melanoma cell colonies was further defined with a dried-slide technique (Salmon & Buick, 1979) and a combination of Papanicolaou and melanin staining (Mishima, 1960). Melanoma colonies from these patients expressed melanin pigmentation, which served as a marker of neoplastic origin of the colony-forming cells (Meyskens *et al.*, 1981).

*Data analysis.*—All assay data were stored on a Wang 2200 C laboratory computer disc file. Cloning efficiencies were calculated from the total number of cells plated, and were not corrected for the proportion of non-tumour cells in the sample. S.e. mean for individual data points averaged 5%. For the figures, the mean of triplicate observations of the survival of tumour colony-forming units (TCFU) for each patient was plotted against drug concentration. Because of practical limits on the number of cultures that can be set up on fresh biopsies, higher plating concentrations were not routinely used to define drug–dose effects at TCFU levels <5% of control.

Previous experience with the assay has suggested that survival of TCFU at one-tenth the calculated pharmacologically achievable concentration × 1h ("cut-off" concentration) provides a useful parameter to relate to clinical response (see Moon *et al.*, 1980, 1981). The "cut-off" concentrations specified were 0·01μg/ml for 1 h for Act D, VDE, and VLB and 0·10 μg/ml for 1h for the remaining drugs. These concentrations were selected on the basis of an extensive compilation of the literature (Alberts & Chen, 1980) and our own pharmacokinetic studies. The doses necessarily represent an approximation to drug concentrations that would be found in an individual patient. On the basis of training sets of *in vitro*/*in vivo* correlations in experiments with ovarian cancer (Alberts *et al.*, 1980*b*) and multiple myeloma (Durie *et al.*, 1981) and on the current study, patients with melanoma were classified as sensitive if survival of TCFU was <38% at the "cut-off" concentration and resistant if >38%. This simple approach to classifying *in vitro* response has developed from a careful analysis of the more complex "area under the curve" (Moon *et al.*, 1980, 1981) and should be viewed as an operational method of determining *in vitro* chemosensitivity and relating TCFU survival to clinical response.

### RESULTS

### *Survival of melanoma TCFU at "cut-off" concentration*

We have examined the effect of a series of drugs on melanoma TCFU. The percentage survivals of melanoma TCFU at the "cut-off" concentration of Act D are profiled in the Figure and summarized in Table I for the 9 drugs tested. The Figure graphically demonstrates the marked heterogeneity of response to a drug of melanoma TCFU, both within a tumour and between tumours, and the frequent presence of a plateau of drug-resistant TCFU even at very high drug concentrations. Using the operational definition of 38% TCFU survival at the "cut-off" concentrations to define *in vitro* sensitivity (Table I), the percentage of tumour stem cells sensitive to the different drugs were: PAM, 36%; ADR, 30%; DDP, 27%; Act D, 26%; AMSA, 20%; VDE, 14%; BCNU, 12%; DTIC, 9%; VLB, 8%.

The effects on melanoma TCFU of two drugs with similar actions were compared. In 4 patients (Nos 22–25) the effect of ADR and AMSA on TCFU was simultaneously available. The TCFU survival at 0·10 μg/ml for ADR *vs* AMSA were (in %) 23 *vs* 19, 30 *vs* 56, 96 *vs* 18, and 111 *vs* 79. The effect of VLB and VDE on TCFU was also simultaneously assessed in 8 patients (No. 33, 38, 30, 42, 45, 47, 48, 49). The percentage TCFU survival at 0·01 μg/ml for VLB *vs* VDE were (in %) 77 *vs* 78, 68 *vs* 67, 104 *vs* 94, 101 *vs* 99, 100 *vs* 101, 77 *vs* 78, 60 *vs* 16, and 81 *vs* 101.

TABLE I.—*Percentage survival of melanoma stem cells at "cut-off" concentration of* 9 *drugs*

| | Drug μg/ml for 1 h | | | | | | | | |
|---|---|---|---|---|---|---|---|---|---|
| Patient | ADR 0·10 | BCNU 0·10 | DDP 0·10 | DTIC 0·10 | PAM 0·01 | VBL 0·01 | Act D 0·01 | VDE 0·01 | AMSA 0·10 |
| 1 | — | **1** | | **21** | — | — | — | — | — |
| 2 | — | — | — | 122 | — | — | — | — | — |
| 3 | 47 | 73 | — | 48 | 56 | 96 | — | — | — |
| 4 | 84 | 84 | — | 46 | — | — | 40 | — | — |
| 5 | 47 | **16** | — | 72 | 46 | — | — | — | — |
| 6 | **31** | 49 | — | — | **27** | — | — | — | — |
| 7 | 52 | 75 | — | **22** | — | — | — | — | — |
| 8 | — | 50 | — | 72 | **33** | — | **32** | — | — |
| 9 | — | 84 | — | 96 | — | — | 45 | — | — |
| 10 | — | 95 | — | 101 | — | — | 104 | — | — |
| 11 | — | **20** | 47 | 110 | 83 | — | 41 | — | 62 |
| 12 | — | 101 | — | 101 | — | — | — | — | — |
| 13 | 56 | — | — | — | — | — | 70 | — | — |
| 14 | — | 67 | 94 | 92 | — | — | **1** | — | **22** |
| 15 | — | — | — | — | — | — | 58 | — | — |
| 16 | — | — | — | — | — | — | 69 | — | — |
| 17 | — | — | — | — | — | — | 92 | — | 76 |
| 18 | — | 68 | — | 94 | 55 | — | 54 | — | 61 |
| 19 | — | — | — | — | — | — | 69 | — | 53 |
| 20 | — | — | — | 64 | — | — | 94 | 90 | 102 |
| 21 | — | — | 94 | — | — | — | — | — | 63 |
| 22 | **23** | 88 | **26** | — | **26** | — | 88 | **19** | **19** |
| 23 | **30** | — | **35** | — | 89 | — | 80 | 95 | 56 |
| 24 | 96 | 51 | — | 78 | 49 | — | 104 | 77 | **18** |
| 25 | 111 | 110 | — | 122 | 97 | — | 91 | 105 | 79 |
| 26 | — | — | 60 | 100 | — | — | — | 88 | **21** |
| 27 | — | — | 46 | — | — | — | **20** | **20** | 85 |
| 28 | — | — | — | — | — | — | **29** | — | 79 |
| 29 | — | 43 | — | **24** | — | — | **20** | 87 | 42 |
| 30 | — | 44 | — | — | — | — | — | **15** | **30** |
| 31 | — | — | 70 | — | — | — | — | 66 | 79 |
| 32 | — | 84 | 101 | 73 | — | — | — | 50 | 86 |
| 33 | — | — | — | 53 | **34** | 77 | **23** | 78 | 94 |
| 34 | — | — | — | — | — | 40 | — | — | — |
| 35 | — | **33** | — | 45 | — | 39 | **6** | — | 42 |
| 36 | — | 86 | — | 74 | — | — | 82 | 89 | 69 |
| 37 | — | 90 | — | 81 | — | — | 78 | **28** | 72 |
| 38 | — | — | — | 60 | — | 68 | 44 | 67 | 104 |
| 39 | — | 72 | — | 46 | — | — | **33** | **33** | 45 |
| 40 | — | 52 | — | 100 | — | 104 | **36** | 94 | 81 |
| 42 | — | — | — | 91 | — | 81 | 79 | — | — |
| 42 | — | 131 | — | 114 | — | 101 | 104 | 99 | 102 |
| 43 | — | 72 | — | — | — | **4** | — | — | — |
| 44 | — | 85 | — | — | — | — | **38** | 99 | — |
| 45 | — | 122 | — | 64 | — | 100 | 89 | 101 | 121 |
| 46 | — | — | **35** | 60 | — | — | **15** | 52 | **15** |
| 47 | — | 65 | — | 81 | — | 77 | 80 | 78 | 94 |
| 48 | — | — | — | — | — | **27** | 87 | **16** | 80 |
| 49 | — | 84 | — | 96 | — | 81 | 54 | 101 | — |
| 50 | — | 94 | 62 | 72 | — | — | 39 | — | — |
| Sensititive* cases | 3 | 4 | 3 | 3 | 4 | 2 | 11 | 6 | 6 |
| Total cases | 10 | 32 | 11 | 33 | 11 | 12 | 36 | 24 | 30 |
| % | 30 | 12·5 | 27 | 9 | 36 | 16·7 | 30·1 | 25 | 20 |

* Sensitive: <38% survival of TCFU.

### *Effect of prior chemotherapy on the* in vitro *sensitivity of melanoma TCFU to drugs*

One hundred and forty *in vitro* drug studies were conducted on TCFU from 20 patients with no prior chemotherapy, and 60 studies were conducted on TCFU from 30 patients with prior chemotherapy (Table II). There was no overall difference in survival of TCFU in response to *in vitro* chemotherapy in those patients who had not received prior chemotherapy (26/140 tests, 19%) and those who had

FIG.—Chemosensitivity of melanoma TCFU to Act D. ○---○ Sensitive *in vitro* (< 38% TCFU survival at "cut-off" concentration of 0·01 μg/ml). ●——● Resistant *in vitro* (> 38% TCFU survival at "cut-off" concentration of 0·01 μg/ml).

TABLE II.—*Effect of prior chemotherapy on the* in vitro *sensitivity of melanoma stem cells to drugs*

| | No prior chemotherapy | | Prior chemotherapy* | | |
|---|---|---|---|---|---|
| Drug | S (%) | R | S (%) | R | *P* (Fisher) |
| ADR | 0 (0) | 6 | 3 (75) | 1 | 0·033 |
| BCNU | 4 (15) | 22 | 0 (0) | 6 | 0·566 |
| DDP | 1 (17) | 5 | 2 (40) | 3 | 0·424 |
| DTIC | 3 (11) | 24 | 0 (0) | 6 | 0·578 |
| PAM | 2 (29) | 5 | 2 (50) | 2 | 0·333 |
| VLB | 1 (13) | 7 | 0 (0) | 5 | 0·666 |
| Act D | 8 (33) | 16 | 3 (25) | 9 | 0·402 |
| VDE | 2 (12) | 14 | 1 (13) | 7 | 0·683 |
| AMSA | 5 (25) | 15 | 1 (10) | 9 | 0·301 |
| Total | 26 (19) | 114 | 12 (20) | 48 | |

* Prior chemotherapy in each instance with agents other than those tested.

(12/60, 20%). There was also no significant difference in survival of TCFU exposed to the 9 individual drugs tested, except for ADR, where prior chemotherapy favoured *in vitro* sensitivity.

*Detection of* in vitro *drug sensitivity of melanoma TCFU*

Discovery of at least one effective drug (< 38% survival of TCFU) for each patient is a desirable goal. We performed 282 *in vitro* trials using the above 9 drugs. Table III summarizes the number of

TABLE III.—*Detection of* in vitro *drug sensitivity of melanoma stem cells**

| Drugs studied | Studies | Studies containing at least 1 active drug† (%) |
|---|---|---|
| 1–2 | 8 | 1 (13)‡ |
| 3–4 | 11 | 6 (55) |
| 5–6 | 26 | 19 (73) |
| 7–8 | 10 | 10 (100) |

* Includes additional patients in which *in vitro/in vivo* correlations were not made.

† < 38% melanoma colony survival at "cut-off" concentration.

‡ Using the test for trend in proportions (Armitage, 1971) there is a significant increase in the proportion of studies in which at least one sensitive drug was found (*P* < 0·0001).

TABLE IV.—*Effect of* in vitro *percentage survival range on* in vitro/in vivo *correlations for Actinomycin D in metastatic melanoma*

| Parameter | | *In vitro/In vivo* correlation* | | | | Correlations of validity† | |
|---|---|---|---|---|---|---|---|
| *In vivo* response‡ | % survival§ (<) | S/S | S/R | R/S | R/R | True positive | True negative |
| 0·83 | 50 | 5 | 4 | 1 | 7 | 0·56 | 0·88 |
| 0·83 | 40 | 5 | 3 | 1 | 8 | 0·63 | 0·89 |
| 0·83 | 35 | 5 | 2 | 1 | 9 | 0·71 | 0·90 |
| 0·66 | 30 | 4 | 1 | 2 | 10 | 0·80 | 0·83 |
| 0·33 | 20 | 2 | 0 | 4 | 11 | 1·00 | 0·73 |

* Correlation: S/S—Cases sensitive *in vitro* and *in vivo*;
S/R—Cases sensitive *in vitro* and resistant *in vivo*;
R/S—Cases resistant *in vitro* and sensitive *in vivo*;
R/R—Cases resistant *in vitro* and *in vivo*. For patients receiving multidrug *in vivo* treatment, the drug with the smallest *in vitro* survival was used to quantitate *in vitro* sensitivity and classify the patient as either *in vitro* sensitive or resistant.

† Validity: True positive equals $\frac{S/S}{S/S+S/R}$; True negative equals $\frac{R/R}{R/R+R/S}$

‡ *In vivo* response equals $\frac{S/S}{S/S+R/S}$

§ % survival of tumour colonies at a "cut-off" concentration of 0·01 μg/ml.

studies and the number of drugs in each investigation in relationship to the number and percentage of instances in which TCFU survival was reduced to < 38% at the "cut-off" concentration. The results show that with more drugs tested there was a significant increase in the proportion of studies in which at least one effective drug was found ($P<0{\cdot}0001$). For a high probability of detecting at least one active drug, at least 6 drugs should be tested in any assay.

### In vitro/in vivo *correlations with Act D*

We have sufficient *in vitro* and *in vivo* clinical data with Act D to begin to assess the relation of *in vitro* sensitivity to this agent to clinical response. The correlation of *in vitro/in vivo* correlations for Act D is shown in Table IV, and includes mixed responses as responses. The *in vivo* response rate was 0·353 (S/S)/(S/S + R/S). Above 35% TCFU survival the predicted *in vivo* response was > 0·83, and fell at lower TCFU survival (which suggested a break-point) particularly since the true positive (S/S)/(S/S + S/R) *in vivo/in vitro* correlation continued to increase with decreasing TCFU survival. In contrast, the true negative (R/R)/(R/R + R/S) *in vitro/in vivo* correlation was maximal (0·90) at ~35% TCFU survival. The extended calculations indicate that 38% TCFU survival gives the best fit of predicted *in vivo* repsnse and true negative *in vitro/in vivo* correlation. These results demonstrate the method by which an acceptable level of correlation for sensitivity (S/S)/(S/S + S/R) or resistance (R/R)/(R/R + R/S) can be determined using percentage survival at the "cut-off" concentration. For example, at 50% TCFU survival predictive accuracy for sensitivity was 56% (5/9) and for resistance 88% (7/8). In contrast, at a 20% TCFU survival the predictive accuracy for sensitivity was 100% (2/2) and for resistance 73% (11/15).

### *Overall experience with clinical correlation*

The *in vitro* melanoma TCFU and *in vivo* clinical response were compared in the 50 melanoma patients treated with a variety of anticancer drugs (Tables V, VI). The 38% *in vitro* "cut off" concentration was derived using the 48 correlations in 41 patients with melanoma. The approach was the same as that detailed above for Act D (Table IV) and was influenced by the observed *in vivo* response rate as well as the true positive and true negative

TABLE V.—*Correlation of* in vitro *melanoma stem-cell survival with clinical response**

| | |
|---|---|
| S/S† | 12 (8) |
| S/R | 7 (11) |
| R/S | 4 (4) |
| R/R‡ | 25 (25) |

* *In vitro* sensitivity is defined as <38% colony survival at $\frac{1}{10}$ the pharmacologically achievable 1h concentration × time. *In vivo* sensitivity is defined as an objective mixed, + partial + complete response. Numbers in parentheses denote the comparisons when mixed clinical responses are classed as progressive disease. S/S, S/R etc. as defined in Table IV.

† True positive rate ((S/S)/(S/S + S/R)) × 100 = 63 (42).

‡ True negative rate ((R/R)/(R/R + R/S)) × 100 = 86 (86).

rates associated with the *in vitro/in vivo* correlations. When a clinical response was defined as a complete, partial, or mixed (see Materials and Methods), reduction in survival of TCFU below 38% correlated with clinical response in 12/19 clinical trials (63%). If mixed responses were regarded as progressive disease, survival of TCFU below 38% correlated with clinical response in 8/19 clinical trials (42%). TCFU survival above 38% correlated with clinical tumour progression in 85% of 29 trials. Using Fisher's exact test, there is a significant association between the *in vitro* and *in vivo* outcome, with $P = 0{\cdot}001$. If mixed responses are excluded in the evaluation, $P = 0{\cdot}06$.

The details of the 12 objective clinical responses are shown in Table VI. In 6 cases the patient had no prior therapy, but in the other 6 the patients had previously received 1–6 drugs (mean = 3). In 4 clinical trials only single agents were used, and in 8 trials more than one agent was used.

### DISCUSSION

It is clear from these studies that human melanoma TCFU exhibit marked heterogeneity in chemosensitivity to the 6 standard agents (ADR, BCNU, DTIC, PAM, VLB, and Act D) and 3 Phase II agents (DDP, VDE, AMSA) tested. Reduction in survival of TCFU to less than 38% at one-tenth the pharmacologically achievable concentration occurred in only 19% of 200 *in vitro* trials with these 9 drugs in the 50 patients studied. These results are, in general, similar to those obtained in our centre for chemosensitivity testing of ovarian cancer (Alberts *et al.*, 1981) and multiple myeloma (Durie *et al.*, 1981). However, in contrast to melanoma TCFU, ovarian and myeloma tumour stem cells which had not been exposed to prior chemotherapy *in vivo* were much more sensitive to chemotherapy *in vitro* (Durie *et al.*, 1981; Alberts *et al.*, 1981). For example, Alberts *et al.* (1981) found significantly more chemosensitivity in untreated patients than in previously treated patients with ovarian cancer for DDP, ADR and bleomycin.

The fact that melanoma cells from untreated patients were no more sensitive to the drugs tested than cells from previously treated patients (Table II) suggest that most melanoma TCFU are inherently resistant to presently available chemotherapeutic agents. Until more effective agents are identified, the acquisition of drug resistance or cross resistance between agents cannot be easily approached in human melanoma, unless serial biopsies were studied from individual patients. The apparent increased sensitivity of melanoma TCFU from patients previously treated with ADR remains unexplained, and merits further laboratory and clinical investigation.

An important goal in the development of an *in vitro* predictive assay is determination of the minimum number of drugs to be tested *in vitro* to identify at least one agent with a high probability of being effective *in vivo*. Our data indicate that the frequency of identifying potentially effective drugs increases with the number of drugs tested per melanoma specimen (Table III). If >6 drugs were tested, a drug producing <38% survival at the "cut-off" concentration was found in all cases (10/10). On the basis of our *in vitro/in vivo* correlations for melanoma (Tables VI, VII) we can predict that patients who are sensitive *in vitro* will

TABLE VI.—*Characteristics of patients with metastatic melanoma whose tumour regressions were predicted by* in vitro *drug assay*

| Patient No. | Previous therapy | *In vitro* R | *In vivo* S | Treatment | Response† | Duration (months) | Response description |
|---|---|---|---|---|---|---|---|
| 46 | None | DTIC<br>VDE | Act D<br>DDP<br>AMSA | DTIC<br>Act D | C | 10+ | Complete disappearance of multiple subcutaneous nodules |
| 13 | DTIC<br>BCNU<br>*HU<br>PAM<br>ADR<br>Act D | ADR<br>Act D | Heat (42°) +DDP | Heat (42°) +DDP | P | 7 | Regression of extensive vaginal and thigh disease |
| 37 | None | DTIC<br>BCNU<br>Act D<br>AMSA<br>HU | VDE | VDE | P | 5+ | > 50% decrease in multiple nodal and skin nodules |
| 48 | BCNU<br>*RT | Act D<br>AMSA | VLB<br>*BLM<br>HU<br>VDE | VLB<br>BLM | P | 5 | > 50% decrease in lung and skin nodules |
| 24 | DTIC<br>BCNU | DTIC<br>BCNU<br>Act D<br>ADR<br>PAM<br>VDE | AMSA | AMSA<br>VDE | P | 3 | > 50% reduction of abdominal mass |
| 14 | DTIC<br>AMSA | | Act D | Act D | P | 2 | > 50% decrease in lung nodules and neck nodules |
| 35 | None | DTIC<br>PAM<br>PALA | BCNU<br>Act D | BCNU<br>Act D | P | 2 | > 50% decrease in lymph nodes |
| 39 | None | BCNU<br>DTIC<br>AMSA | Act D<br>VDE | DTIC<br>Act D | P | 2 | > 50% decrease in multiple lung and skin nodules |
| 1 | None | None | DTIC<br>BCNU | DTIC<br>BCNU | M | 3 | > 50% decrease in axillary nodes; inguinal nodes stable |
| 22 | BCNU<br>DTIC<br>HU<br>Act D<br>*TX<br>VDE | BCNU<br>Act D | ADR<br>AMSA<br>DDP<br>PAM<br>VDE | AMSA | M | 1 | > 50% decrease in liver mass; skin progression |
| 27 | BCNU<br>DTIC<br>Act D<br>TX | AMSA<br>*MGBG<br>DDP | VDE<br>Act D | AMSA<br>VDE<br>DDP | M | 2 | > 50% reduction of tongue mass, progression of subcutaneous nodules |
| 30 | DTIC | BCNU<br>*PALA | AMSA<br>VDE | AMSA<br>VDE | M | 1 | > 50% decrease in liver metastases; lung nodules stable |

* HU: hydroxyurea; RT: radiotherapy; TX: tamoxifen; BLM: bleomycin.
MGBG: methyl glyoxal bis (guanyl hydrazone). PALA: N-(phosphonoacetyl(-L-aspartate.
† C: complete; P: partial; M: mixed.

respond 40–60% of the time. Since the overall clinical response rate of metastatic melanoma to the best single agents is 10–15% (Einhorn *et al.*, 1974) and to the best combination chemotherapy is 30–35%, this approach may be useful for drug selection in patients with metastatic melanoma.

The conceptual and practical steps involved in determining *in vitro* TCFU

sensitivity and obtaining clinical correlations has been extensively reviewed elsewhere (Salmon, 1980; Moon *et al.*, 1980; 1981; Alberts *et al.*, 1981). Initially, a training set of *in vitro* drug–survival curves and clinical-response data was analysed, and clinical sensitivity and resistance were related to an area under the survival curve with increasing drug concentrations *in vitro* (Moon *et al.*, 1980; 1981). Pharmacological considerations provided further understanding of this system. Reduced survival of TCFU at low drug concentrations (<10% of the pharmacologically achievable 1h concentration for drugs with a short half-life) *in vitro* proved important for identifying ranges of drug concentrations relevant for predicting clinical response. This system has been further refined and operationally simplified, such that if the pharmacology of a drug is reasonably well known, *in vitro/in vivo* correlations can be simply related to percentage survival of TCFU at the "cut-off" concentration. The use of this latter approach is seen in Table IV for Act D and metastatic melanoma. The acquisition of further data for specific tumours and drugs will allow further refinement. With presently available information, <38% colony survival has served as a useful demarcation between clinical sensitivity and resistance. It is important to emphasize that with further *in vitro/in vivo* experience this discrimination point may change.

Except for the higher frequency of mixed responses in melanoma, the correlation of *in vitro* TCFU survival and clinical response for metastatic melanoma is very similar to our results for ovarian cancer (Alberts *et al.*, 1980; 1981) multiple myeloma (Durie *et al.*, 1981), and a variety of other tumours (Salmon *et al.*, 1980), an observation which has been confirmed by other groups (Von Hoff *et al.*, 1981; Mann *et al.*, 1981).

Important corroborations of our findings have recently been reported in studies of human melanoma xenografts studied both *in vivo* in immunodeficient mice and *in vitro* in agar culture (Courtenay & Mills, 1978; Fodstad *et al.*, 1980; Bateman *et al.*, 1980; Tveit *et al.*, 1980; Selby & Steel, 1981). However, important differences between our results and these investigators exist. Our PE is usually in the range 0·01–0·1%, whilst the PE in xenografts and agar diffusion chambers has been closer to 1–5%. We have also frequently seen plateaus in colony survival with increasing drug concentrations, while a linear decrease in colony survival has been recorded in the other systems. There are innumerable possible explanations for these differences. The most important consideration is to determine the nature of the "resistant" colonies in the plateau. This plateau may be real and may represent a subfraction of resistant clonogenic cells which are not grown in the other systems. Alternatively, the plateau may represent clonogenic cells which have received sublethal damage and are destined to die if allowed to replicate further. The plateau "survivors" do not represent aggregates, as they are not present within 24 h of plating, but developed and grew in the agar and are not morphologically distinguishable from melanoma colonies on control plates. To directly assess the reason for the plateau will require replating of dispersed cells from the resistant colonies in the absence and presence of the drug.

In our studies, sensitivity or resistance to agents such as DTIC *in vitro* corresponded with efficacy of the drug *in vivo* as well. Both our studies and those of Tveit *et al.* (1980) with human melanoma xenografts, indicate that DTIC activity *in vitro* is an indicator of clinical response. Previously, there had been one report suggesting that *in vivo* bioactivation was required for DTIC (Loo *et al.*, 1976); this now appears less likely.

Overall, our investigations provide a quantitative description of drug sensitivity of human metastatic melanoma TCFU to a series of agents, with somewhat more success than we anticipated for a tumour that is generally considered drug resistant. Additionally, as has also been the case with other neoplasms, the assay is quite accurate (86%) in melanoma for identifying drugs to which the tumour will be resistant. From the clinical standpoint, prospective identification of drug resistance can spare patients needless expense and toxicity from useless agents. The data also suggest: (a) that meaningful clinical responses are more likely to be associated with a TCFU survival below 38% at low drug concentrations, and (b) minimally active drugs may be identified by accepting higher levels of survival of TCFU. These studies also demonstrate that drug resistance in human melanoma TCFU is often present before any drug exposure *in vivo*. These observations imply that melanoma TCFU are frequently resistant to presently available drugs, and that either new classes or new modalities of therapy will be needed to treat melanoma.

We thank B. Soehnlen, K. Wilde and J. Leibowitz for technical assistance, R. Markmann for secretarial assistance, J. Isaman for computer graphics and the following physicians for referral of patients: G. Altschuler, M. Boxer, M. Chassin, P. Duffey, G. Giordano, R. Jackson, S. Ketchel, R. Ligorsky, E. Lipp, N. Mansour, P. McFarland, R. Rothman, J. Rubach, D. Steinway, T. Wasserman and A. Wendt.

Supported in part by Public Health Service Grants (CA17094, CA21839 and CA23074) and a grant from the American Cancer Society (PDT 184).

### REFERENCES

ALBERTS, D. S. & CHEN, H. S. G. (1980) Tabular summary of pharmacokinetic parameters relevant to *in vitro* drug assay. In *Cloning of Human Tumor Stem Cells* (Ed. Salmon). New York: A. Liss, Inc. p. 351.

ALBERTS, D. S., CHEN, H. S. G., SALMON, S. E. & 4 others (1981*a*) Chemotherapy of ovarian cancer directed by the human tumor stem cell assay. *Cancer Chemother. Pharmacol.* (in press).

ALBERTS, D. S., SALMON, S. E., CHEN, H. S. G., MOON, T. E., YOUNG, L. & SURWIT, E. A. (1981*b*) Pharmacologic studies of anticancer drugs using the human tumor stem cell assay. *Cancer Chemother. Pharmacol.* (in press).

ALBERTS, D. S., SALMON, S. E., CHEN, H. S. G. & 4 others (1980) Predictive chemotherapy of ovarian cancer using an *in vitro* clonogenic assay. *Lancet*, ii, 340.

BATEMAN, A. E., SELBY, P. J., STEEL, G. G. & TOWSE, G. D. W. (1980) *In vitro* chemosensitivity tests on xenografted human melanoma. *Br. J. Cancer*, **41**, 189.

COURTENAY, V. D. & MILLS, J. (1978) An *in vitro* colony assay for human tumours grown in immune-suppressed mice and treated *in vivo* with cytotoxic agents. *Br. J. Cancer*, **37**, 261.

DURIE, B. G. M., YOUNG, L. A. & SALMON, S. E. (1981) Human myeloma stem cell culture: Relationship between *in vitro* drug sensitivity, kinetics, and patient survival duration. *Blood* (in press).

EINHORN, L. H., BURGESS, M. A., VALLEJOS, C. & 8 others (1974) Prognostic correlations and response to treatment in advanced metastatic malignant melanoma. *Cancer Res.*, **34**, 1995.

FODSTAD, O., AASS, N. & PIHL, A. (1980) Response to chemotherapy of human, malignant melanoma xenografts in athymic, nude mice. *Int. J. Cancer*, **25**, 453.

HAMBURGER, A. W. & SALMON, S. E. (1977) Primary bioassay of human tumor stem cells. *Science*, **197**, 461.

LOO, T. L., HOUSEHOLDER, G. E., GERULATH, A. H., SANDERS, P. H. & FARQUHAR, D. (1976) Mechanism of action and pharmacology studies with DTIC (NSC-45388). *Cancer Treat. Rep.*, **60**, 149.

MANN, B. C., STORM, F. K., KERN, D. M., GIVLIANO, A. & MORTON, D. L. (1981) Predictive value of the clonogenic assay in the treatment of melanoma with DTIC and DTIC hyperthermia. *Proc. Am. Soc. Clin. Oncol.*, **22**, C-389.

MEYSKENS, F. L., SOEHNLEN, B. J., SAXE, D. F., CASEY, W. J. & SALMON, S. E. (1981) *In vitro* clonal assay for human metastatic melanoma cells. *Stem Cells*, **1**, 61.

MISHIMA, Y. (1960) Modification of combined DOPA-premelanin reaction. New technique for comprehensive demonstration of melanin, premelanin, and tyrosinase sites. *J. Invest. Dermatol.*, **34**, 355.

MOON, T. E., SALMON, S. E., CHEN, H. S. G. & ALBERTS, D. S. (1980) Quantitative association between *in vitro* and *in vivo* studies. In *Cloning of Human Tumor Stem Cells* (Ed. Salmon). New York: Liss & Co. p. 209.

MOON, T. E., SALMON, S. E., CHEN, H. S. G. & ALBERTS, D. S. (1981) Quantitative association between *in vitro* and *in vivo* studies. *Cancer Chemother. Pharmacol.* (in press).

SALMON, S. E. (Ed.) (1980) *Cloning of Human Tumor Stem Cells.* New York: Liss & Co.

SALMON, S. E., HAMBURGER, A. W., SOEHNLEN, B., DURIE, B. G. M., ALBERTS, D. S. & MOON. T. E. (1978) Quantitation of differential sensitivity of human tumor stem cells to anticancer agents. *N. Engl. J. Med.*, **298**, 1321.

SALMON, S. E., ALBERTS, D. S., DURIE, B. G. M. & 5 others (1980*a*) Clinical correlations of drug sensitivity in the human tumor stem cell assay. *Recent Results Cancer Res.*, **74**, 299.

SALMON, S. E., MEYSKENS, F. L., ALBERTS, D. S., SOEHNLEN, B. & YOUNG, L. (1981) New drugs in ovarian cancer and malignant melanoma: *In vitro* Phase II screening with the human tumor clonogenic cells assay. *Cancer Treat. Rep.*, **65**, 1.

SALMON, S. E. & BUICK, R. N. (1979) Preparation of permanent slides of intact soft-agar colony cultures of hematopoietic and tumor stem cells. *Cancer Res.*, **39**, 1133.

SALMON, S. E., ALBERTS, D. S., MEYSKENS, F. L. & 6 others (1980*b*) Clinical correlations of *in vitro* drug sensitivity. *Cloning of Human Tumor Stem Cells* (Ed. Salmon). New York: Liss, Inc. p. 223.

SELBY, P. J. & STEEL, G. G. (1981) Clonogenic cell survival in cryopreserved human tumor cells. *Br. J. Cancer*, **43**, 143.

TVEIT, K. M., FODSTAD, O., OLSNES, S. & PIHL, A. (1980) *In vitro* sensitivity of human melanoma xenografts to cytotoxic drugs: Correlation with *in vivo* chemosensitivity. *Int. J. Cancer*, **26**, 717

VON HOFF, D. O., CASPER, J., BRADLEY, E., JONES D. & MAKUCH, R. (1981) Association between human-tumor colony forming assay results and response of an individual patient's tumor to chemotherapy. *Am. J. Med.*, **70**, 1027.